# A brief history of brain iron accumulation in Parkinson disease and related disorders

**DOI:** 10.1007/s00702-022-02505-5

**Published:** 2022-05-09

**Authors:** Paul B. Foley, Dominic J. Hare, Kay L. Double

**Affiliations:** 1 Medical Journal of Australia, Sydney, Australia; 2 Atomic Medicine Initiative, University of Technology, Sydney, Australia; 3 Brain and Mind Centre and School of Medical Sciences (Neuroscience), Faculty of Medicine and Health, University of Sydney, Sydney, Australia

**Keywords:** Parkinson disease, iron, substantia nigra, history of neuroscience

## Abstract

Iron has a long and storied history in Parkinson disease and related disorders. This essential micronutrient is critical for normal brain function, but abnormal brain iron accumulation has been associated with extrapyramidal disease for a century. Precisely why, how, and when iron is implicated in neuronal death remains the subject of investigation. In this article, we review the history of iron in movement disorders, from the first observations in the early twentieth century to recent efforts that view extrapyramidal iron as a novel therapeutic target and diagnostic indicator.

## Introduction

Satisfying the high energy demands of the brain requires adequate access to iron because of its essential role in electron transport and ATP production (Lill et al. 2012). Iron is also needed for numerous biochemical processes specific to the central nervous system; for example, as a cofactor in myelin synthesis by oligodendrocytes (Todorich et al. 2009). Iron deficiency during critical developmental windows consequently impairs the elaboration of neural networks and cell signaling pathways, resulting in neurodevelopmental deficits (Gerlach et al. 1994; Beard and Connor 2003; Lozoff and Georgieff 2006; Hare et al. 2013a).

The key feature of iron in many chemical reactions is its propensity to donate electrons under physiological conditions. Redox cycling—whereby ferrous iron (Fe^2+^) is oxidized to ferric iron (Fe^3+^), which in turn is reduced to the ferrous state—allows the catalysis of hundreds of repeated reactions by relatively low concentrations of iron (micrograms per gram tissue). But the reactivity of iron can also be detrimental: in the Fenton reaction (first described in 1894: Fenton 1894), part of the Haber‒Weiss reaction (Haber and Weiss 1934), Fe^2+^ catalyzes the generation of cytotoxic hydroxyl radicals from hydrogen peroxide produced during mitochondrial respiration (Meneghini 1997). To maintain iron homeostasis in the brain, a complex network of regulatory proteins and signaling pathways constrain the impact of these deleterious reactions, and antioxidant proteins (e.g., superoxide dismutase 1, glutathione peroxidases) and other antioxidant species (e.g., α-tocopherol, coenzyme Q10) mitigate cellular damage (Hare et al. 2013a). A variety of transporter, chaperone, and storage proteins transport iron into and around the brain, ensuring delivery to where it is needed, while levels of unbound intracellular iron (primarily as Fe^2+^), the “labile iron pool”, are kept low by minimizing iron import, shunting iron excess to storage proteins (e.g., ferritins), and promoting its export (Kakhlon and Cabantchik 2002; Moos et al. 2007).

**Fig. 1:**
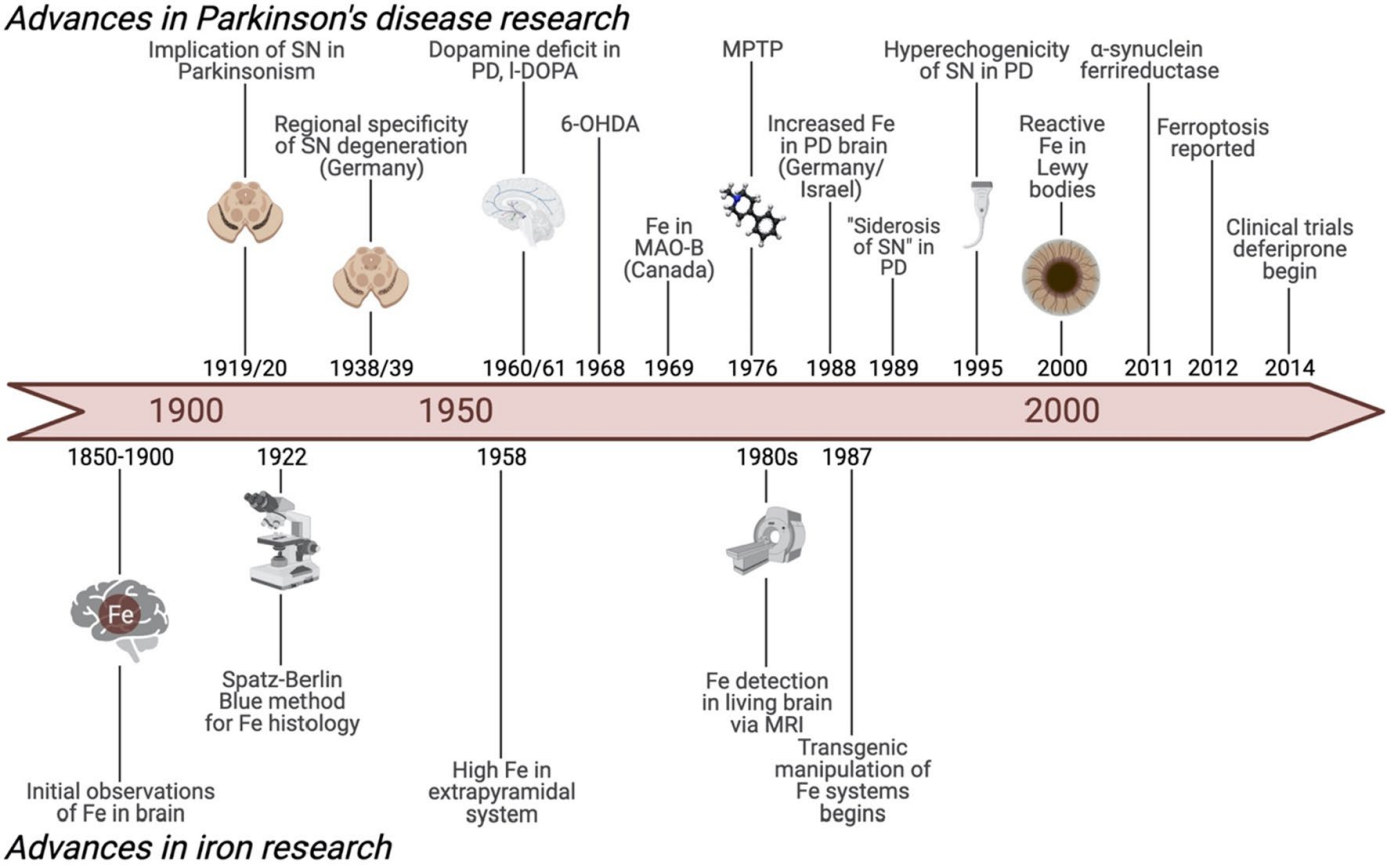
Timeline of some of the major developments in iron and Parkinson disease (PD) research

In this article, we outline the history of iron in Parkinson disease and other neurodegenerative disorders. Oxidative stress caused by iron dyshomeostasis is a driving factor in many neurodegenerative diseases (Ward et al. 2014; Valko et al. 2016), and it is also invoked as a major component of the free radical theory of aging (Schipper 2004). A role for iron metabolism in the pathophysiology of parkinsonism has an even longer history (Fig. 1), stretching back a century to the time of another pandemic that captured international attention. Whether a change in iron metabolism is a cause or consequence of parkinsonism, however, remains unresolved; the answer to this question is critical to how iron biochem-istry is viewed in the context of developing novel therapies for Parkinson disease and related movement disorders.

## 1880‒1950: Initial observations

The presence of non-hemoglobin iron in the brain (i.e., iron other than that bound in erythrocytes), albeit at much lower levels than in other organs, had been recognized since the late nineteenth century. In 1887, Polish chemist Stanisław Szczepan Zaleski (1858‒1923; Dorpat) found that iron reactions in the brain (using ammonium sulfide or potassium ferrocyanide) were much stronger in gray than white matter (Zaleski 1887). More specifically, Romanian neuroanatomist Gheorghe Marinescu (1863‒1938) noted in 1909 that hemosiderin (degraded ferritin‒iron complexes) and melanin coexisted in some substantia nigra cells, but that melanin itself was iron-free (Marinesco 1909). In 1914, Italian pathologist Giosuè Biondi (1885–1959) described iron particles in neuronal nuclei and neuroglia of the substantia nigra and other tegmental mesencephalic regions in arteriosclerotic dementia, as well as in the cytoplasm of neurons, glia, and adventitial cells of the pallidum (Biondi 1914).

In 1915, Italian pathologist Pietro Guizzetti (1862–1937) visualized granules of ‘masked iron’—tissue-bound ferric iron, released by treatment with acid alcohol—in unfixed brain slices, using Max Perls’ 1867 Berlin (Prussian) blue method. In higher mammals, the reaction was strongest in the globus pallidus, substantia nigra, nucleus dentatus, and (in humans only) the nucleus ruber. He described a diffuse iron reaction in vascular walls, the neuronal cytoplasm, and the nucleus and cytoplasm of glia in these regions, and fine cytoplasmic granules around but never within the nuclei. Guizzetti further noted that brain iron was not present in younger animals; in humans, the reaction was first evident in the pallidum at about six months and peaked at eight years of age, while in the nigra the response commenced at 9‒12 months, and peaked at 16 years of age (Guizzetti 1915). German pathologist Otto Lubarsch (1866‒1933) reported that iron deposits in the neuroglia of the striatum, substantia nigra (but not co-located with melanin), and pituitary reflected local processes for removing erythrocytes that had leaked through the particularly thin walls of the vasculature in these regions (Lubarsch 1917).

**Fig. 2:**
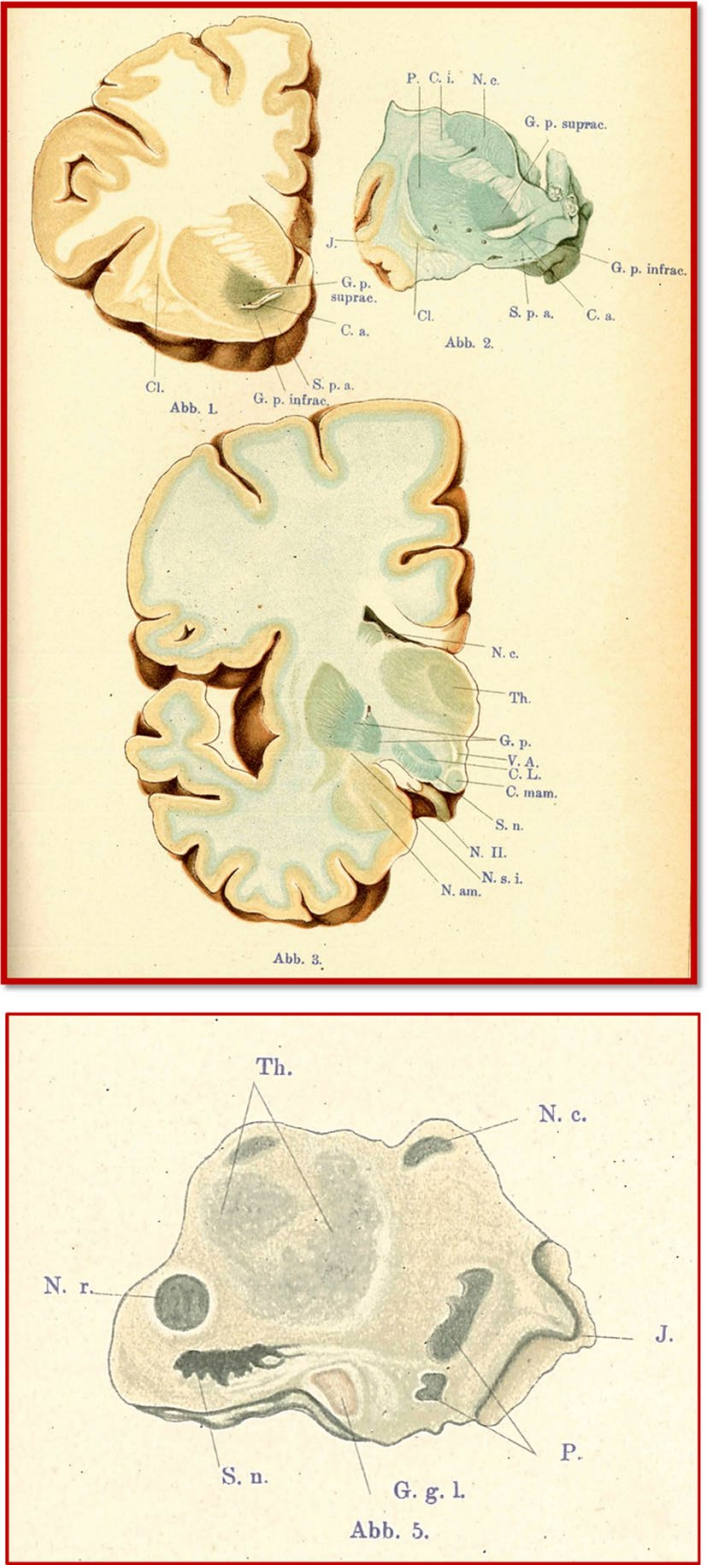
Depiction by Spatz (1922a, b) of macroscopic distribution of iron staining in coronal sections of unfixed human brain, visualized with the ammonium sulfide method. Abb. 1. 42-year-old woman, section through rear left frontal lobe; concentrated ammonium sulfide for one minute: supra- and infra-commissural pallidum gray, otherwise no reaction. Abb. 2. 16-year-old boy, slightly caudal to plane of Abb. 1; ½ hour in 2% ferrocyanide solution, 15 h in dilute hydrochloric acid. Abb. 3. 62-year-old man, section through medial mammillary body; ¼ hour in 2% ferrocyanide solution, ¼ hour in hydrochloric acid. The blue coloration of the white matter is more intensive in the image than in reality. Abb. 5. 37-year-old woman, section through the caudal basal ganglia; several days in ammonium sulfide in alcohol. The strongest reaction is in the substantia nigra, then the nucleus ruber, the nucleus caudatus, and the caudal part of the putamen (the pallidum is not included in this section). Cl. = claustrum; C. L. = corpus Luysi; C. m. = corpus mamillare; C. a. = commissura anterior; C. i. = capsula interna; G. p. = globus pallidus (infrac. = pars infracommissuralis; p. suprac. = pars supracommissuralis); G. g. l. = ganglion geniculatum laterale; J. = lnsula; N. c. = nucleus caudatus; N. am. = nucleus amygdalae; N. r. = nucleus ruber; N. II. = nervus opticus; N. s. i. = nucleus substantiae innominatae; P. = putamen; S. n. = substantia nigra; Th. = thalamus; S. p. a. = substantia perforata anterior; V. A. = Vicq d’Azyr tract

In Germany, the Munich-based neuroanatomist Hugo Spatz (1888‒1969) interpreted these deposits quite differently. Spatz examined brain iron with the ammonium sulfide method for his macroscopic (Fig. 2) and the Turnbull Berlin blue method for his microscopic analysis, culminating in 1922 in a 130-page article accompanied by colored plates that garnered broader attention than Guizzetti’s report, overshadowed by the Great War. He could only assess the ephemeral iron reaction qualitatively, but two brain region groups were distinguished by particularly strong iron reactions:

1. The pallidum and the substantia nigra reticulata (but not the melanin-containing pars compacta): the reaction was stronger in glia than in neurons.
2. The red nucleus, striatum, dentate nucleus, and subthalamic nucleus: a primarily diffuse reaction, although the striatum also included iron granules.

In contrast to Lubarsch, Spatz distinguished the ‘endogenous’ or ‘functional iron’ in these regions from the ‘meta-bolic iron’ derived from erythrocyte decay; it was not transported by the accumulating cells, but was required for local metabolic processes, including the recently elucidated cellular respiration pathways. The iron-containing centers were structurally quite diverse in location and structure, but connected by pathways implicated in the regulation of muscle tone. Spatz therefore proposed “uniting these centers and their connective tracts as an extrapyramidal motor system” (Spatz 1922b). This was the first systems model based on biochemical relationships in the brain, and the earliest attempt to realize the concept recently proposed by the prominent neuroanatomists Cécile and Oskar Vogt (Berlin) that a regional chemical or metabolic characteristic might be critical to the function of that region, or could predispose it to dysfunction (“pathoclisis”) (Vogt and Vogt 1922).

Spatz conceived his model during the height of the encephalitis lethargica pandemic in Europe, an infection that elicited a variety of motor symptoms, including acute and chronic parkinsonism. Shortly after he submitted his synthesis for publication (August 1921), the Frankfurt-based neurologist Kurt Goldstein (1878–1965) reported his preliminary findings on the nigral lesion in chronic encephalitis lethargica (Goldstein 1921), unaware that Konstantin Trétiakoff (Paris) had described similar findings two years earlier (Trétiakoff 1919). Spatz recognized the significance of both reports for his contentious inclusion of the substantia nigra in the extrapyramidal motor system and his view that the nigral lesion underlay the parkinsonism of chronic encephalitis lethargica (Spatz 1922a,b).

Shortly thereafter, neuropathologist Julius Hallervorden (1882–1965) consulted Spatz regarding a woman who had had severe motor symptoms before her death at the age of 24 years. The only macroscopic change in the brain was the rust-brown coloration of the substantia nigra reticularis and pallidum, which they attributed to disturbed iron metabolism in glial cells, consistent with “the assumption of increased demand as well as stagnation in the operation of oxidative processes” (Hallervorden and Spatz 1922). The first biochemical change identified in an extrapyramidal disorder was thus localized increases in reactive iron levels. The genetic disorder suffered by the woman and several other members of her family was known as “Hallervorden‒Spatz disease” from the late 1930s, and more recently as pantothenate kinase-associated neurodegeneration (PKAN). It is one type of “neurodegeneration with brain iron accumulation”, a group of at least a dozen inherited metabolic disorders characterized by iron deposits in the basal ganglia, with a variety of neurologic and psychiatric symptoms, including progressive dementia, that generally emerge during late childhood and early adolescence (D'Mello and Kindy 2020).

Spatz found that iron levels were increased in encephalitis lethargica in the glia (but not the neurons) of the substantia nigra reticulata and the medial pallidum; in more advanced disease, iron clumps also appeared in the oligodendroglia of the reticulata. Similar changes were seen in Huntington disease (mainly in the striatum) and schizophrenia (dementia praecox) (Metz and Spatz 1924). Spatz’ colleague August Metz (1878–1945) observed that the increased iron content was initially accumulated by oligodendroglia, as fine granules; only later were the microglia recruited, accumulating iron as coarse granules, and still later the astrocytes near blood vessels (Metz and Spatz 1926). Spatz interpreted this distribution as reflecting a local “disparity between supply and demand”: microglia ( an only recently recognized cell type) were forced to accept the overflow that resulted from neuronal loss and their consequent loss of activity.

Spatz’ macroscopic findings on brain iron were particularly positively received. In 1923, they were quantitatively confirmed by Otto Wuth (1885–1946) (Wuth 1923), and Dutch pathologist Abraham Gans (1885–1971) commented that a “reaction that demonstrates specific chemical properties of certain centers of the brain cannot fail to be of interest to the pathologist. The iron reaction does that with wonderful clarity” (Gans 1923).

Increased nigral iron levels were also sometimes reported in non-extrapyramidal system disease (e.g., in neurosyphilis: Struwe 1928) or outside the extrapyramidal system in people with extrapyramidal disease (e.g., in the frontal cortex in post-encephalitic parkinsonism, although much more marked in the substantia nigra: Kingo 1934).

In 1923, German pathologist Friedrich Lewy (1885–1950) confirmed Spatz’ findings regarding iron localization, but particularly noted the perivascular iron deposits in the pallidum and striatum of brains from people with Parkinson disease (paralysis agitans) (Lewy 1923). Vascular wall iron deposits, particularly in the pallidum, had been described in a number of toxic and other pathologic conditions during the previous quarter century, but the Romanian neuroanatomists Marinesco and Draganesco emphasized that “active, atomic” iron could be identified in most neurons if appropriate methods were used (Marinesco and Draganesco 1923).

**Fig. 3:**
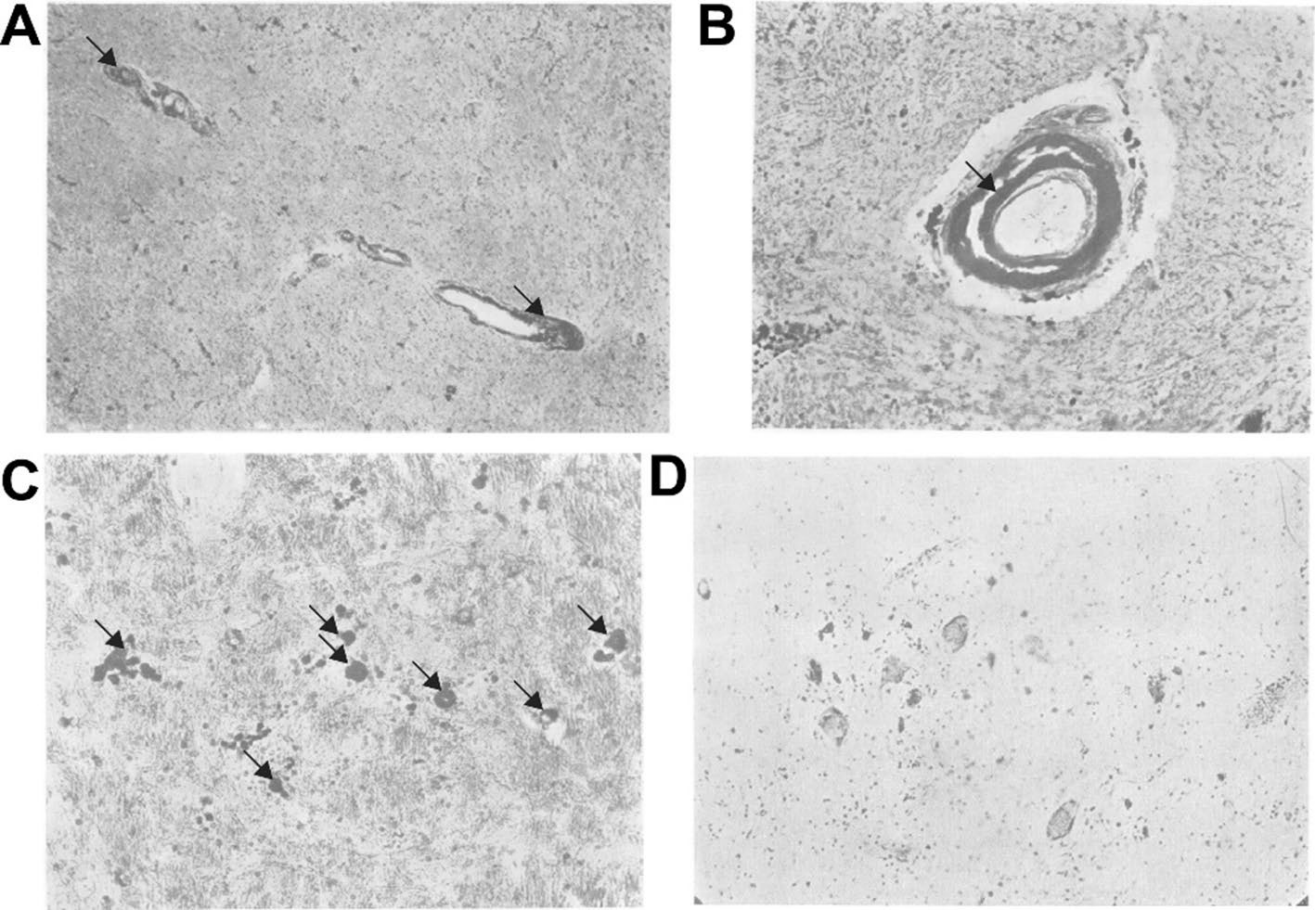
An early histopathological assessment of iron in the Parkinson disease brain using Perls Prussian blue staining described by Lhermitte et al. (1924). **a**, **b** Deposition of non-heme iron was found in globules lining the vessel walls of the globus pallidus (arrows; both with hematoxylin and Van Giesen stain; ×60 magnification for (**a**); ×180 for (**b**). **c** These deposits were also observed as extracellular globules with a high fat content (arrows; Scharlach R and hematoxylin stain; ×200 magnification) in globus pallidus tissue. **d** The substantia nigra showed no abnormal iron deposition, though pigmented cells were noted to be shrunken (Nissl stain; ×140 magnification). Figures reproduced with permission from BMJ Publishing.

Using Perls staining, Jean Lhermitte (Paris), Walter Kraus (New York) and Douglas McAlpine (London) reported in 1924 that intracellular iron levels were diminished in the pallidum (but normal in the substantia nigra) in post-encephalitic parkinsonism, replaced by abnormal vascular wall deposits and siderophilic globules (Fig. 3). Although consistent with earlier findings in parkinsonism, the authors cautiously concluded that they had "no proof that these deposits play any part in the symptomatology of the disease" (Lhermitte et al. 1924).

Iron-related pathology was also reported in individual cases of other parkinsonian disorders. In 1932, abnormal deposits in the pallidum were described in a 24-year-old man with “progressive pallidal degeneration”, characterized by parkinsonism and rigidity (Winkelman 1932); the disorder was perhaps akin to progressive supranuclear palsy, also marked by iron deposits in the basal ganglia and nigra (Dexter et al. 1991; Boelmans et al. 2012). Prominent Boston neurologist Stanley Cobb (1887‒1968) wrote in 1932 that “in … any degeneration or inflammation of the basal ganglia, [pallidal siderosis] is enhanced and iron rings may be found about the vessels of young people” (Cobb 1932). In 1935, two Indiana physicians described a 60-year-old laborer who developed severe parkinsonian symptoms after falling eight feet from a pile of rubber; autopsy two and a half years later found marked neural loss and glial and microglial iron accumulation in the nigra (Turnbull blue staining) (Bruetsch and DeArmond 1935). On the other hand, another study found normal levels of detectable iron in two cases of post-encephalitic parkinsonism (Bahr 1935), and a comprehensive summary of parkinsonism case studies to 1942 included only one (post-encephalitic) in which (pallidal) iron deposits were described (Benda and Cobb 1942).

Würzburg pathologist Martin Benno Schmidt (1863‒1949) concluded in 1940 that “Spatz’ interpretation that [iron] was connected with the activities and metabolism of the centers in which it is located—namely, those of the extrapyramidal motor system—is completely justified” (Schmidt 1940). Nevertheless, interest in whether iron was involved in the pathophysiology of parkinsonism had largely lapsed by this point for want of a conceptual framework, and attention gradually shifted to the Lewy bodies (first described by Friedrich Lewy in 1912, and so designated in 1919 by Trétiakoff), with which changes iron levels were not associated (Greenfield and Bosanquet 1953).

## 1950‒1980: Revival of interest in extrapyramidal system iron

As late as the 1980s, Spatz’ macroscopic findings were cited as the major publication on the subject, and confirmed by investigators using improved versions of the Perls reaction (Meguro et al. 2007). The Swedish investigators Hallgren and Sourander confirmed in 1958 that iron levels in the human brain were highest in extrapyramidal system structures; levels increased during the first two decades of life, but plateaued by 30 years of age. About one-third of non-heme iron was bound to a ferritin-like protein that the authors assumed was an iron store reserved for the specific needs of the brain (Hallgren and Sourander 1958). The marked concentration of iron in some regions of the extrapyramidal basal ganglia circuit vulnerable to Parkinson disease, including the substantia nigra, is now recognized (Davies et al. 2013), and is a feature of the human brain absent in other species such as the mouse (Hare et al. 2012a, b).

Alfonso Asenjo (1906‒1980) and colleagues at the Neurosurgical Institute in Chile undertook a series of light and electron microscopy studies of brain iron during the second half of the 1960s. They identified abnormal iron deposits in neurons and glia in ventrolateral thalamic tissue from four patients with various types of parkinsonism; siderosis was also evident in *post mortem* samples from various peripheral organs, but not in the cerebral cortex (Rojas et al. 1965). However, blood, urine and cerebrospinal fluid iron levels were similar in people without and without parkinsonism (Asenjo 1968). The authors subsequently found that chronic administration of large doses of iron to rabbits and monkeys (100 mg/week i.v., for up to 50 weeks) increased iron levels in the basal ganglia (Oberhauser et al. 1970) and elicited “parkinsonian” symptoms (primarily tremor) (Aranda and Asenjo 1969). Charles Still (South Carolina) similarly proposed in 1977 that a porous blood–brain barrier and “positive body iron balance” could lead to parkinsonism resulting from “brain iron overload” (Still 1977).

American neuropathologist Kenneth Earle (1919–1996; Washington), using X-ray fluorescence spectroscopy (which detects iron in biological tissue with very high sensitivity and spatial resolution (Pushie et al. 2014), found that that iron levels in brains from eleven people with parkinsonism were twice those in controls, and that the difference was greater in gray than white matter (Earle 1968). The spatial distribution of iron-rich granules in the putamen in ‘striato-nigral degeneration’ (now: multiple system atrophy) was visualized by Arnulf Koeppen and colleagues (New York) with electron probe X-ray microscopy and Perls staining in 1971 (Koeppen et al. 1971). Each of these studies used tissue fixed with formalin, a process long known to leach iron from biological tissue (Gōmōri 1936).

Only scattered reports of iron status in people with parkinsonism were otherwise published before 1980; for instance, slightly elevated CSF level in one of two patients with parkinsonism (Kjellin 1967), but normal serum and urinary levels of iron, transferrin, and ferritin (Campanella et al. 1973). However, major advances in knowledge about iron metabolism during this period would later have significant implications for Parkinson disease research, including studies of the iron storage protein ferritin (Dognin and Crichton 1975) and ferroxidase proteins such as ceruloplasmin (Curzon and O'Reilly 1960), although ceruloplasmin was not linked with parkinsonism until several decades later (Ayton et al. 2013). It was also during this period that the role of iron in monoamine oxidase activity was first examined by Ted Sourkes, Moussa Youdim and colleagues in Montreal (Symes et al. 1969), with later implications for dopamine metabolism.

Further, neurotoxins which cause relatively specific lesions of the nigrostriatal pathway became available that later served in laboratory models of parkinsonism, including 6-hydroxydopamine (6-OHDA) in 1968 (Ungerstedt 1968) and 1-methyl-4-phenyl-1,2,3,6-tetrahydropyridine (MPTP) in 1976 (Langston and Palfreman 2014). It would later prove that their neurotoxicity involve iron-mediated oxidative stress (reviewed: Blum et al. 2001; Hare et al. 2013b; Hare and Double 2016); 6-OHDA itself has also been characterized as a neurotoxic metabolite of dopamine formed by iron-catalyzed autoxidation (Kienzl et al. 1999). In 1985, Judes Poirier, John Donaldson, and André Barbeau (Montreal) proposed that high levels of transition metals, including iron, in the substantia nigra could explain the specificity of the damage wreaked by MPTP (Poirier et al. 1985).

The exact role of iron in MPTP toxicity is still unclear, as is its relevance to the natural pathogenesis of idiopathic Parkinson disease. MPTP is metabolized by iron-containing monoamine oxidase B (MAO-B) in astrocytes to 1-methyl-4-phenylpyridinium (MPP^+^), and elevated brain iron levels may exacerbate this process; increasing brain iron levels by dietary means during periods of brain development indeed potentiates MPTP toxicity (Kaur et al. 2007). Nevertheless, the link between MPTP and MAO-B activity spurred the initial use of MAO-B inhibitors (e.g., selegiline, rasagiline) as therapy for people with Parkinson disease (Jenner and Olanow 1996), although the basis of their clinical utility is now thought to be much more complex.

## 1980‒2000: Part 1. Building a case for iron and neurodegeneration

New quantitative measures of total iron, as well as separate assessment of the two redox states of iron provided new insights into the role of iron in parkinsonism. It had long been recognized that quantifying tissue iron using traditional staining techniques was subject to several limitations; not only was it specific for ferric iron, including that bound by ferritin and hemosiderin, but the technique inevitably leached iron from the assayed tissue samples (Gōmōri 1936).

In 1988, Peter Riederer and colleagues, including Emin Sofič, Wolf-Dieter Rausch, Paul Kruzik (Vienna) and Moussa Youdim (Haifa), reported that total substantia nigra iron levels quantified by spectrophotometry were increased by 176% and iron(III) levels by 255% in fresh frozen tissue samples from eight people with parkinsonism, while levels in the cortex, hippocampus, putamen, and globus pallidus were similar to those in age-matched controls; the authors suspected that the specificity of the change compared with earlier reports might be explained by age differences in the source patients (Sofic et al. 1988). Further, they identified an inversion of the iron (II)/iron (III) ratio in the substantia nigra (from 2:1 to 1:2) (Riederer et al. 1989). Riederer and his colleagues interpreted their findings as possibly providing “an indirect indication of enhanced oxidative processes” (Sofic et al. 1988). They later specified the pars compacta as the site of greatest change (Sofic et al. 1991). Riederer and colleagues also reported that nigral ferritin levels were increased in parkinsonism (Riederer et al. 1989; Jellinger et al. 1990), but both unchanged and reduced levels were also documented (reviewed: Mochizuki et al. 2020).

At about the same time, David Dexter, Peter Jenner and colleagues (London), using inductively coupled plasma spectroscopy, reported a more modest 31‒35% increase in nigral iron and 29% decrease in the pallidum (in frozen tissue) (Dexter et al. 1987, 1989). Further, Jenner and colleagues found a generalized reduction in ferritin levels throughout the brain, including in the substantia nigra, in parkinsonism, but not progressive supranuclear palsy or multiple system atrophy, which also feature increased total iron levels (a response to neurodegeneration in affected basal ganglia regions) and nigral cell death (Dexter et al. 1991).

The Riederer and Jenner groups were both cautious about the implications of their findings for the pathophysiology of parkinsonism. The increase in brain iron levels with normal aging (Markesbery et al. 1984), however, was consistent with abnormal accumulation being involved in diseases such as parkinsonism, in which age is the major risk factor, and the significance of increased ferric iron levels for local oxidative stress were clear (Götz et al. 1990).

On the other hand, Ali Rajput and colleagues in Canada, for instance, found that levels of iron (and most other metals) assessed with atomic absorption and emission were near normal in fixed substantia nigra tissue from parkinsonian patients (Uitti et al. 1989); Loeffler and colleagues (Detroit) concurred, but found increased levels in the pallidum (Loeffler et al. 1995). In particular, investigators who employed Mössbauer spectroscopy generally did not find significant increases in total iron levels in parkinsonism (Friedman and Galazka-Friedman 2012), a pattern that may be explained by the relatively low accuracy of Mössbauer spectroscopy (Gerlach et al. 1995; Hare et al. 2012a, b).

Discordance regarding substantia nigra iron levels in parkinsonism (and, indeed, differing estimates of the normal levels) may have been related to the tissue assessed (fixed or unfixed), sample treatment, the detection methods applied, and the age and disease state of the source patients. In general, however, both semi-quantitative and quantitative analyses have concluded that total iron levels in the nigra are increased in parkinsonism; changes in other regions in *post mortem* tissue have been less consistent (reviewed: Sian-Hülsmann et al. 2011; Hare et al. 2012a, b; Ayton and Lei 2014).

Early findings regarding the association of iron with neuromelanin were also discordant. Electron microscopy with X-ray microprobe analysis indicated that iron levels were increased in neuromelanin-free areas of fixed substantia nigra (that is, sites of neurodegeneration) and reduced in melanin clumps; they were particularly high in Lewy bodies, which also included aluminum, and the authors concluded that the high iron levels, not found in progressive supranuclear palsy, were not attributable solely neurodegeneration (Hirsch et al. 1991). However, Daniel Perl and colleagues, using a laser microprobe mass analyzer to assess fixed tissue, found that iron levels of intra-neuronal neuromelanin granules were higher than in the non-melanized neuronal cytoplasm or adjacent neuropil, and higher in parkinsonism than control tissue (Good et al. 1992).

In 1989, Youdim, Dorit Ben-Shachar, and Riederer proposed that Parkinson disease results from a “progressive siderosis” of the substantia nigra involving oxidative stress driven by an iron–neuromelanin interaction (Youdim et al. 1989). Direct imaging of metal association with neuromelanin later confirmed that this pigment avidly binds ferric iron (as well as other metals) and that increased levels of iron are present on neuromelanin in the substantia nigra in Parkinson disease (Jellinger et al. 1992; Gerlach et al. 2003; Bohic et al. 2008). In a healthy cellular environment, neuromelanin, by binding iron at high- and low-affinity binding sites (Double et al. 2003), appears to effectively sequester excessive iron in vulnerable neurons (Li et al. 2005), but when its buffering capacity is exhausted by the abnormal accumulation of cellular iron it may actively contribute to the redox-active iron pool and thereby to oxidative stress-induced neurodegeneration (reviewed: Hare and Double 2016; Mochizuki et al. 2020; Sian-Hulsmann and Riederer 2021).

In 2001, Zecca and colleagues reported that nigral total iron levels (in contrast to those of the locus ceruleus) increased during the first four decades of life and then remained stable into old age (90 years); ferritin (light and heavy chain) levels also increased across life (also different to the locus ceruleus), but were lower than those of neuromelanin, which increases and matures with age (Zecca et al. 2001a; Fedorow et al. 2006). Mössbauer spectroscopy indicated that the major iron(III) storage in nigral neurons was neuromelanin (Zecca et al. 2001b; Friedman and Galazka-Friedman 2012). Werner and colleagues found in 2008 that nigral ferritin levels were increased (light and heavy chain ferritins, but statistically significant only for heavy chain ferritin (Werner et al. 2008), while Koziorowski reported in 2007 that nigral levels of light chain ferritin were lower and those of heavy chain ferritin higher in Parkinson disease (Koziorowski et al. 2007). Friedman and Galazka-Friedman have interpreted such findings as indicating that altered ferritin shell structure and the consequent leakage of free iron are more important for the pathophysiology of parkinsonism than total iron levels (Friedman and Galazka-Friedman 2012).

In 2000, reactive iron was identified in Lewy bodies (Castellani et al. 2000), the inclusion bodies, composed largely of α-synuclein, that are typical of idiopathic Parkinson disease. Both iron(II) and iron(III) can bind to α-synuclein, but iron(III) particularly favors α-synuclein aggregation and fibril formation, and reactive oxygen species generation (reviewed: Zecca et al. 2001b).

**Fig. 4:**
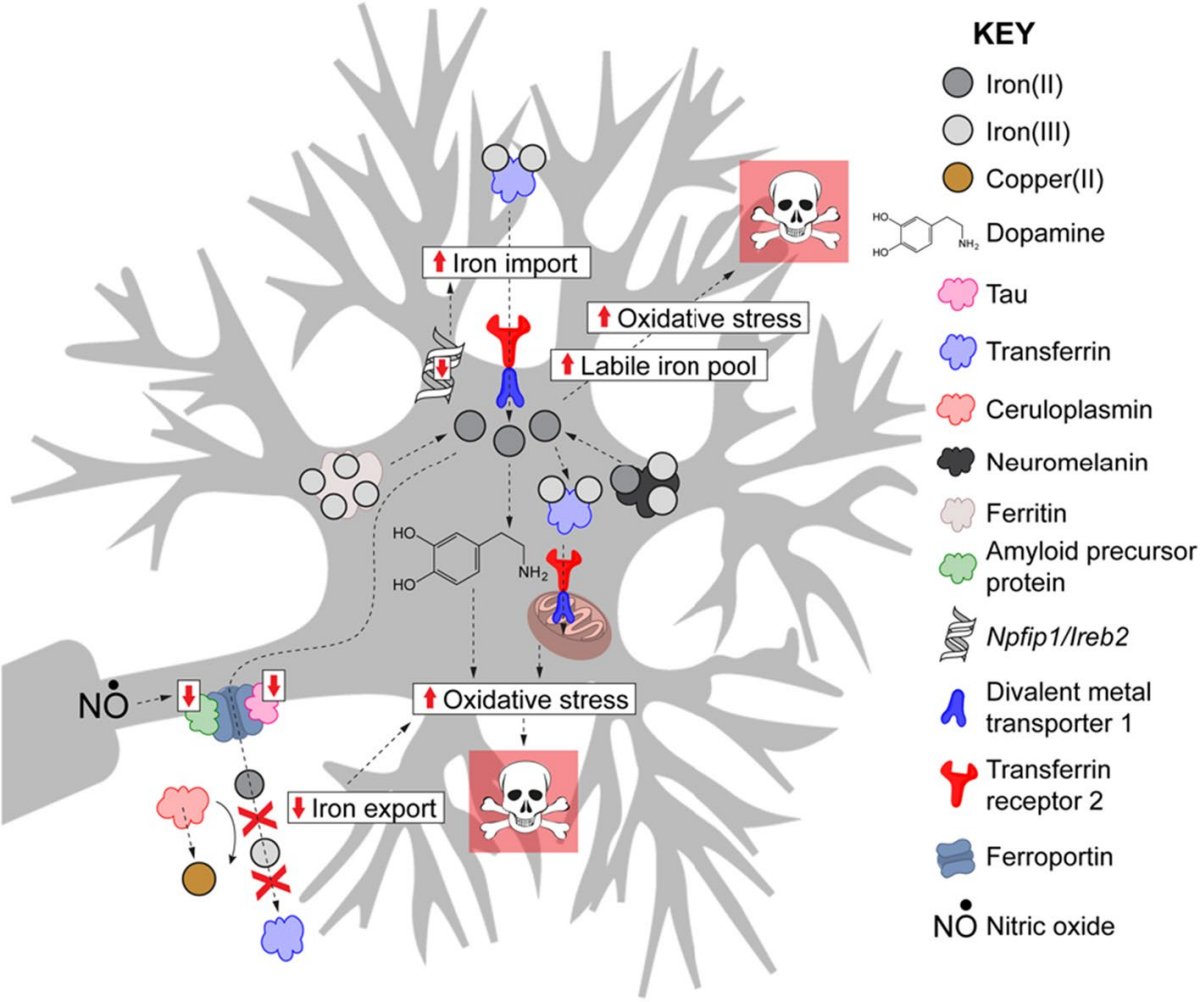
Iron metabolism in Parkinson disease. Proposed mechanism of iron accumulation in dopaminergic neurons and sources of oxidative stress. Reproduced with permission from Hare and Double, 2016

Mutations in several iron-related proteins have been associated with risk of Parkinson disease, including ferritin, transferrin, iron-regulatory protein 2, and divalent metal transporter 1 (reviewed: Hare et al. 2013a; Fig. 4). Peripheral iron metabolism, on the other hand, was generally found to be unrelated to the risk of parkinsonism. A meta-analysis of all quantitative reports of iron in the substantia nigra and biofluids in Parkinson disease concluded that cerebrospinal iron levels were non-significantly higher and serum/plasma levels somewhat lower in parkinsonism, while CSF and serum/plasma ferritin and transferrin and serum/plasma lactoferrin and haptoglobin concentrations are similar in people with Parkinson disease and controls (reviewed: Jiménez-Jiménez et al. 2021).

## 1980‒2000: Part 2. New technologies, new discoveries: the in vivo imaging revolution

**Fig. 5:**
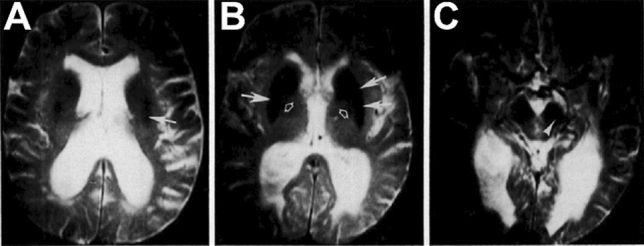
Early T2-weighted magnetic resonance imaging using a 1.5 T field strength of iron in a multiple system atrophy. White arrows in **a**, **b** denote decreased signal intensity, indicating iron deposits, in the putamen and caudate nucleus compared to the globus pallidus (open arrows, (**b**)). Hypointensity marked with the white arrowhead (**c**) was interpreted as indicative of specific iron accumulation in the substantia nigra pars compacta. Reproduced from Drayer et al. 1986a with permission from the Radiological Society of North America (copyright 1986)

Interest in the role of iron in the pathophysiology of parkinsonism was boosted by recognition of the ability of magnetic resonance imaging (MRI), developed during the 1970s and introduced into the clinic in the 1980s, to detect and quantitate iron in the brains of living patients, particularly the substantia nigra. The paramagnetic properties of brain non-heme iron cause local areas of magnetic field inhomogeneity that reduce transverse relaxation times (T_2_) (Drayer et al. 1986b). In early studies, however, MRI findings were only incompletely correlated with *post mortem* reports based on Perls staining for iron. Initial imaging studies of living patients, some investigators found that nigral iron levels were reduced in parkinsonism (Rutledge et al. 1987), while others found iron deposits in the putamen, caudate, and substantia nigra compacta (Fig. 5) in patients with multisystem atrophy or progressive supranuclear palsy (Drayer et al. 1986b). Subsequent studies with larger numbers of patients (Antonini et al. 1993) and stronger magnetic fields (Gorell et al. 1995) confirmed that areas of T_2_-weighted hypointensity were typical in the nigra, caudate, and putamen in parkinsonism, but *"*shortening of T_2_ values in the substantia nigra did not correlate with disease duration nor with clinical severity*"* (Antonini et al. 1993). That is, nigral iron deposits were an early feature of parkinsonism, consistent with their playing a causative role in its pathogenesis, but also with their being unrelated to the disease process altogether.

The accuracy and specificity of in vivo brain iron measurements increased as MRI technology improved during the 1990s, allowing the two alternatives to be more closely examined. For instance, George Bartzokis (1956–2014) employed two MRI systems with different magnetic field strengths (0.5 and 1.5 T) to measure the field-dependent increase in R_2_ (the inverse of T_2_), a specific measure of total iron in the mineral core of ferritin molecules (Bartzokis et al. 1993). Bartzokis and his colleagues found evidence for increased ferritin-bound iron in people with early onset disease in the nigra compacta and reticulata, putamen and pallidum, but reduced ferritin capacity in the nigra reticulata in people with later onset Parkinson disease. The authors concluded that their results “suggest that dysregulation of iron metabolism occurs in Parkinson disease and that this dysregulation may differ in earlier- versus later-onset Parkinson disease” (Bartzokis et al. 1999). Substantia nigra compacta R_2_ values have subsequently been found to be significantly higher in recently diagnosed parkinsonism and to gradually increase with disease progression (reviewed: Feraco et al. 2021). The importance of the development of technologies capable of quantifying iron in the living central nervous system was underlined by the accruing data indicating that iron levels, and the status of iron-regulatory proteins, in the periphery do not reflect brain iron levels in Parkinson disease (Genoud et al. 2020; Jiménez-Jiménez et al. 2021).

**Fig. 6:**
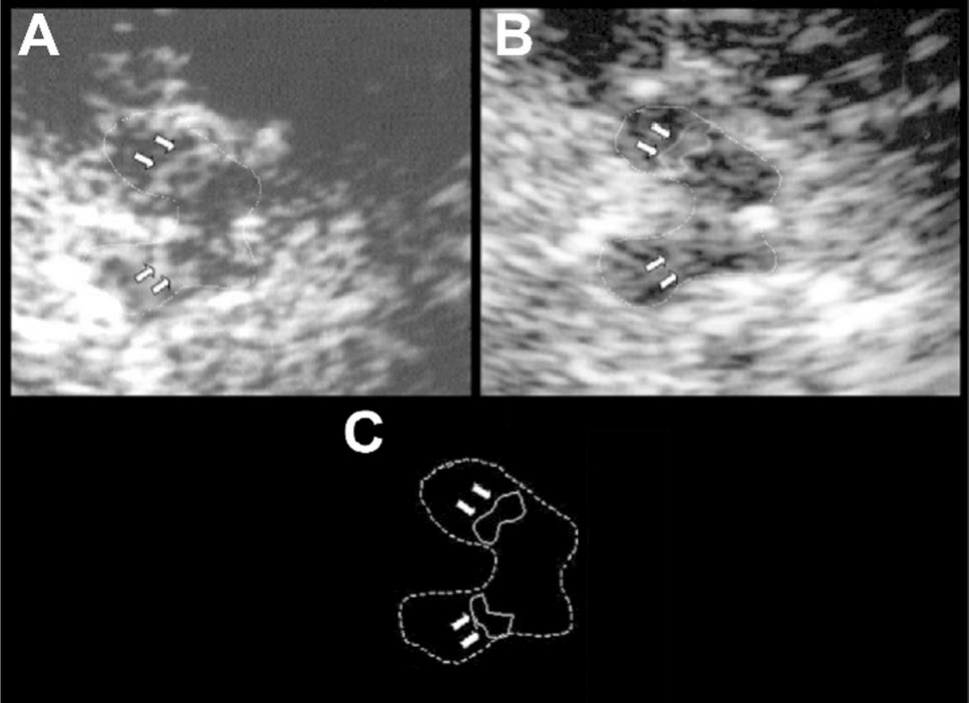
Transcranial ultrasonogram showing progressive hyperechogenicity of the substantia nigra pars compacta in Parkinson disease. **a** Initial examination revealed an area of 0.23 cm^2^ hyperechogenicity in the substantia nigra pars compacta (white arrows). **b** Follow-up examination at five years showed hyperechogenicity had not changed. **c** Schematic representation of the ipsilateral mesencephalon, with the substantia nigra pars compacta marked with white arrows. Reproduced from (Berg et al. 2005) with permission from John Wiley and Sons (copyright 2005)

Transcranial ultrasonography was also applied to assessing brain iron in the mid-1990s. Hyperechogenicity of the substantia nigra, viewed through the temporal bone window, is increased in Parkinson disease. First reported in 1995 by Georg Becker and colleagues (Würzburg), it was initially attributed to nigral gliosis and regarded as being correlated with disease severity and duration (Becker et al. 1995). Daniela Berg, who had detected nigral iron accumulation and increased echogenicity in 6-OHDA-treated rats (Berg et al. 1999b), confirmed that hyperechogenicity was a cost-effective means for screening for the iron accumulation in the basal ganglia (Berg et al. 1999a, 2002; Berg 2009). She and her colleagues also found that nigral hyperechogenicity developed early—indeed, before the emergence of any symptoms (Berg et al. 2011)—and did not change in the course of the disease (Berg et al. 2005; Fig. 6), leading to transcranial ultrasonography being included in the Movement Disorders Society research criteria for identifying people at increased risk of Parkinson disease (Berg et al. 2015).

Magnetic resonance imaging of brain iron is now an integral tool in Parkinson disease research (Pyatigorskaya et al. 2014). It may also be of clinical value in the future: for instance, one study found that iron changes were restricted to the ventral nigra over a period of three years, the regions that degenerates earliest and most completely in Parkinson disease (Bergsland et al. 2019). Further, nigral iron load in people with Parkinson disease may be correlated with motor disability, as measured by UPDRS rigidity and freezing of gait scores (reviewed: Heim et al. 2017; Mochizuki et al. 2020; Feraco et al. 2021). The degree of nigral iron elevation generally reflects the severity of motor symptoms, but not the duration of disease (Wallis et al. 2008).

New imaging protocols, including susceptibility-weighted imaging and quantitative susceptibility mapping (QSM), have significantly improved image resolution, allowing even more specific regional measurements. A meta-analysis of brain iron levels in Parkinson patients, assessed by MRI and *post mortem* histochemical analysis, confirmed the substantia nigra compacta as the site of most marked iron accumulation, with significant but less marked accumulation in the putamen, red nucleus, caudate nucleus, and pallidum in later stages of the disease (Wang et al. 2016). QSM iron findings, in particular, have been found to reflect both disease condition and duration, including lateral asymmetry of iron deposition in the compacta and the spread of iron pathology in later disease; further, it distinguishes between the patterns of this pathology in different types of parkinsonism (reviewed: Mochizuki et al. 2020; Ryman and Poston 2020; Feraco et al. 2021). Both the R_2_ and QSM modalities produce nigral iron data that are correlated with post mortem assessment, but α-synuclein aggregation could also be assessed with the R_2_ approach (Lewis et al. 2018). More recently, a technique for simultaneously imaging iron and neuromelanin as complementary imaging biomarkers has offered a tantalizing opportunity to investigate their interacting roles in the physiopathology of Parkinson disease, particularly during its early stages (He et al. 2021).

## 2001–2010: The transgenic toolbox opens

In the first decade of the new century, discussions of the role of iron in the etiology of Parkinson disease, particularly its role in stimulating oxidative stress and its potential as a target for treatment, continued (for instance:Berg et al. 2001; Ke and Ming Qian 2003; Götz et al. 2004; He et al. 2021). Further reports of changes in iron-associated pathways in brain tissues in Parkinson disease advanced knowledge in this field, but captured only a single time point in the course of the disease, usually late stage disease, and were possibly also subject by post mortem changes. Other studies of iron systems in biofluids from living Parkinson patients could be collected from early stage patients, but their relevance to changes in the central nervous system remains unclear (Genoud et al. 2020). Chemically induced animal models of Parkinson disease are still valuable, but the possibility of new murine models have excited interest as technologies for targeted genetic modifications have advanced. The first insertion of transgenic material into mice embryos was achieved in 1974, and the passage of implanted genes to offspring in 1981. In 1987, the first method for genetically ablating genes was reported (reviewed: Saunders 2020), completing a suite of tools required for further probing the roles of iron-regulatory proteins suspected of playing important roles in the pathogenesis of Parkinson disease (Gerlach et al. 1994).

Initial studies focused more on the broad effects of response factors to increased oxidative stress, such as the overexpression of human superoxide dismutase-1 (SOD1) or deletion of mitochondrial superoxide dismutase-2 (SOD2) (Chan et al. 1995). Mice that overexpress SOD1 were resistant to MPTP toxicity (Przedborski et al. 1992), echoing earlier studies that indicated MPTP intoxication was linked with increased oxidative stress. The oxidative stress hypothesis of Parkinson disease was investigated using a range of interventions thought to reduce cellular oxidative load, including the antiparkinsonian drug apomorphine, to investigate their effects on MPTP-induced dopamine neuron death (Grünblatt et al. 1999). The development of sophisticated strains of genetically modified mice later allowed the expression or knockout of genes involved in iron regulation, such as ferritin, ceruloplasmin, and tau protein, in specific neuron populations, including tyrosine hydroxylase-positive neurons, facilitating the investigation of their effects on iron-associated neuron damage (Kaur et al. 2003; Thompson et al. 2003; Zhu et al. 2010; Lei et al. 2012; Ayton et al. 2013). The production of mice expressing human wildtype of mutant forms of α-synuclein also provided new ways to study the role of this important protein in the etiology of the synucleinopathies (Kahle et al. 2001; Hashimoto et al. 2003). In 2011, it was reported that α-synuclein acts as a cellular ferrireductase (Davies et al. 2011), a function later shown to be impaired in Parkinson disease (McDowall et al. 2017), adding another iron-associated pathway to the Parkinson story. Two years later, a deficiency of the soluble form of the microtubule-associated protein tau, linked with the pathology of Alzheimer disease but also commonly deposited in the parkinsonian brain, was found in a transgenic mouse study to result in toxic iron accumulation in the brain, suggesting that the toxic properties of this protein may involve its interaction with iron (Lei et al. 2012).

## 2011–2021: The translation of discovery science

The wealth of information linking iron and Parkinson disease, carefully collected and analyzed over more than 120 years, led to the idea that therapies which maintain brain iron levels within the normal range might have a practical neuroprotective effect (Lange et al. 1994). The hypothesis was tested *in vitro* as early as 1990 (Tanaka et al. 1990), and later in mouse models of Parkinson disease; for example, following chemical lesions that produce parkinsonism-relevant dopamine neuron loss (e.g., Gal et al. 2010). By the turn of the twenty-first century, promising findings stimulated discussion in major international journals of whether modulating brain iron might be the next direction in anti-parkinsonian therapy, possibly for the first time modifying the course of the disease (Kaur et al. 2003). The discovery of a new iron-dependent cell death pathway, ferroptosis (Dixon et al. 2012), and the identification of this pathway in Parkinson disease-relevant models (Zhang et al. 2020) further stimulated interest in this area. In the early 2000s, the ability of clioquinol, an antifungal hydroxyquinoline with metal-chelating properties, to modify central iron levels in Parkinson disease was investigated (Kaur et al. 2003). But while preclinical findings were promising, links between clioquinol and a sensory neuropathy disorder (Egashira and Matsuyama 1982) undermined interest in its potential. It was later reported that the moderate iron chelator deferiprone, used to treat people with the iron overload disorder thalassemia, was neuroprotective for catecholaminergic neuroblastoma cells exposed to MPTP (Molina-Holgado et al. 2008) and in the 6-OHDA model of Parkinson disease (Dexter et al 2011), and it also improved motor function in a mouse model of synucleinopathy (Carboni et al. 2017). Deferiprone was also reported to reverse abnormal iron deposition in Friedrich ataxia (Abbruzzese et al. 2011) and possibly also  in PKAN (Klopstock et al. 2019).

In 2014, David Devos in Lille reported the results of a pilot double-blind, placebo-controlled clinical trial of deferiprone in patients with early stage Parkinson disease: after six months’ treatment, their motor performance improved and the frequency of adverse events was reduced. Further, the R2* MRI parameter in the substantia nigra (thought to reflect iron load) was significantly reduced, and it increased after treatment stopped (Devos et al. 2014; Grolez et al. 2015). The success of this pilot study prompted more advanced trials of deferiprone (FAIRPARKI and FAIRPARKII), the results of which are not yet available (University Hospital Lille 2016). Meanwhile, David Dexter, continuing at Imperial College London his long term work on iron in Parkinson disease, found in a small clinical trial that deferiprone reduced brain iron levels in people in Parkinson disease (Martin-Bastida et al. 2017). The success of these trials has led to interest in applying this approach to other degenerative disorders in which iron accumulation is a feature; for example, a pilot trial of deferiprone for treating amyotrophic lateral sclerosis found that it reduced iron levels in the brain and spinal cord, with modest but positive clinical benefits (Moreau et al. 2018).

## Conclusion

That the iron economy of the extrapyramidal system is abnormal in parkinsonism has been established. How the described changes arise, and how they relate to the pathophysiology of parkinsonism, however, remain matters of debate (Fig. 5). Possible reasons for local elevations in iron level include age- or disease-dependent loss of iron storage protein capacity, increased importation (increased expression of transferrin receptor 1 and divalent metal transporter 1) or reduced export (reduced expression of ferroportin-1) in Parkinson disease, and microglial activation in response to neurodegeneration. Whether the increased levels are the cause or a consequence of neuronal loss, iron-associated degenerative pathways may play roles in driving progressive neurodegeneration (reviewed: Gerlach et al. 2006; Hare et al. 2013a; Ma et al. 2021; Riederer et al. 2021).

Plausible mechanisms of harm include iron-dependent programmed cell death (ferroptosis) and increased misfolding and aggregation of α-synuclein. α-Synuclein oligomers can activate both apoptosis via calcium ion influx and ferroptosis by iron-dependent reactive oxygen species production and lipid peroxidation reduce iron(III) to iron(II) using copper as a cofactor and NADH as electron donor; and activate microglia with all its consequences. Further, α-synuclein mRNA has a structured iron-responsive element that controls translation that is activated at higher iron concentrations (reviewed: Sian-Hulsmann and Riederer 2021).

More than 120 years after iron was first identified in the brain, its importance for the etiology of Parkinson disease is recognized. Information on the nature of this phenomenon, carefully collected over many years by laboratory researchers, and enabled by a series of technical developments that have underpinned their studies, are now being harnessed by researcher–clinicians testing the thesis that modifying central iron levels may offer hope for that most elusive of treatments: a disease-modifying therapy for people with Parkinson disease.
